# Supplementary material for: The interactome of intact mitochondria by cross-linking mass spectrometry provides evidence for coexisting respiratory supercomplexes
Source: Mol Cell Proteomics. 2017 Dec 8;17(2):216–32. doi: 10.1074/mcp.RA117.000470 (PMC5795388; doi:10.1074/mcp.RA117.000470)
Supplement: Supplemental Data [file supp_RA117.000470_133922_0_supp_23978_fzffwf.zip › spectra_annotation/mito_DR_spectra_annotation/431-1-16-1-13-1.pdf]

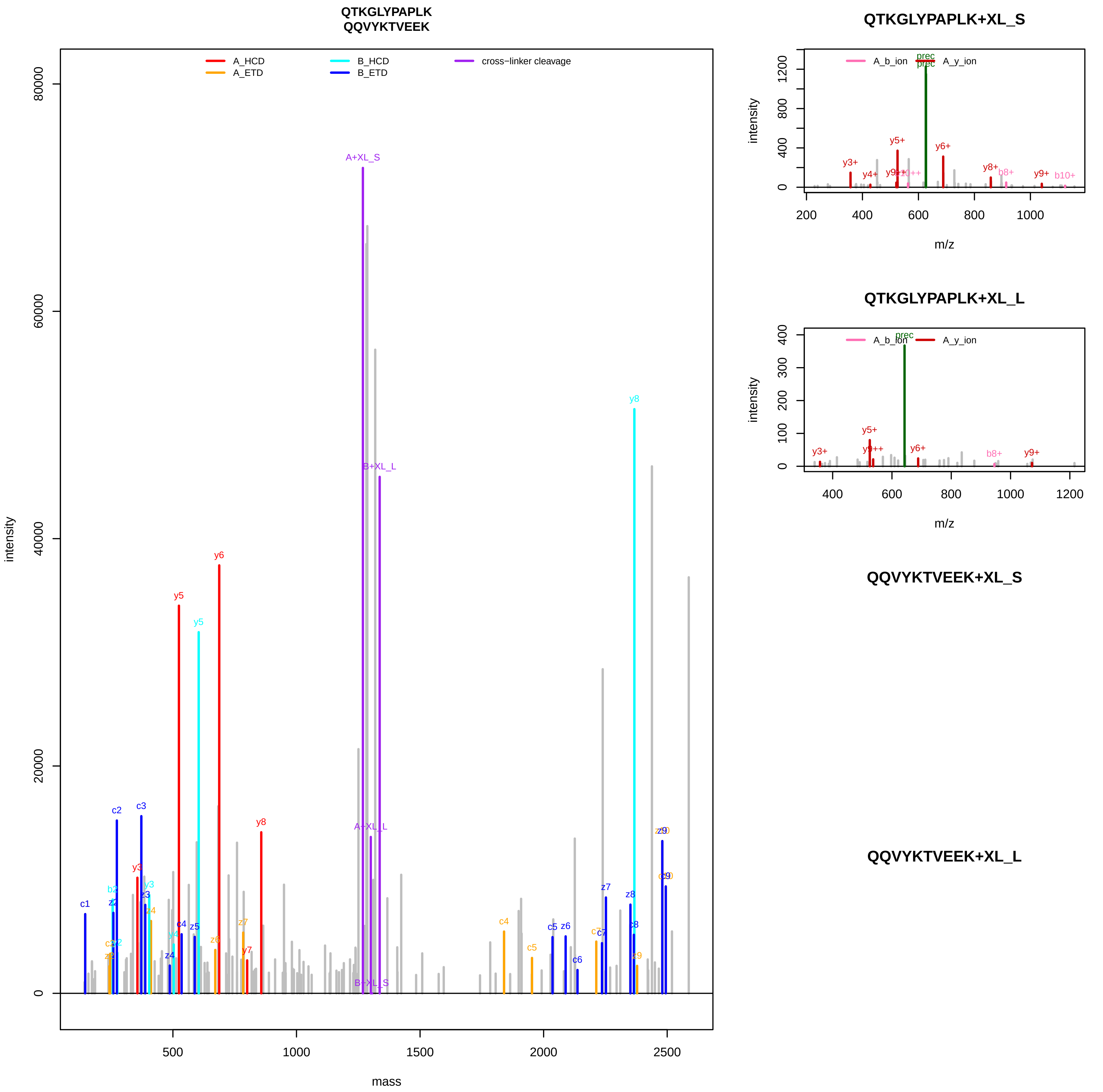
QTKGLYPAPLK
QQVYKTVEEK
A_HCD
A_ETD
B_HCD
B_ETD
cross−linker cleavage
A+XL_S
B+XL_L
A+XL_L
B+XL_S
y5
y6
y8
y3
y7
c1
c2
c3
z3
c4
z4
z5
z6
z7
c4
c5
c6
c7
z8
c8
z9
c9
intensity
mass
QTKGLYPAPLK+XL_S
A_b_ion
A_y_ion
prec
y3+
y4+
y5+
y6+
y8+
b8+
y9+
b10+
m/z
QTKGLYPAPLK+XL_L
QQVYKTVEEK+XL_S
QQVYKTVEEK+XL_L
